# TADILOF: Time Aware Density-Based Incremental Local Outlier Detection in Data Streams

**DOI:** 10.3390/s20205829

**Published:** 2020-10-15

**Authors:** Jen-Wei Huang, Meng-Xun Zhong, Bijay Prasad Jaysawal

**Affiliations:** Department of Electrical Engineering, National Cheng Kung University, Tainan City 701, Taiwan; s1993126@gmail.com (M.-X.Z.); bijay@jaysawal.com.np (B.P.J.)

**Keywords:** outlier detection, local outlier factor, data streams, air quality monitoring

## Abstract

Outlier detection in data streams is crucial to successful data mining. However, this task is made increasingly difficult by the enormous growth in the quantity of data generated by the expansion of Internet of Things (IoT). Recent advances in outlier detection based on the density-based local outlier factor (LOF) algorithms do not consider variations in data that change over time. For example, there may appear a new cluster of data points over time in the data stream. Therefore, we present a novel algorithm for streaming data, referred to as time-aware density-based incremental local outlier detection (TADILOF) to overcome this issue. In addition, we have developed a means for estimating the LOF score, termed "approximate LOF," based on historical information following the removal of outdated data. The results of experiments demonstrate that TADILOF outperforms current state-of-the-art methods in terms of AUC while achieving similar performance in terms of execution time. Moreover, we present an application of the proposed scheme to the development of an air-quality monitoring system.

## 1. Introduction

The expansion of Internet of Things is increasing the importance of outlier detection in streaming data. A wide range of tasks ranging from factory control charts to network traffic monitoring depend on the identification of anomalous events associated with intrusion attacks, system faults, and sensor errors [1,2]. Some outlier detection methods are designed to find global outliers, while some methods try to find local outliers [1,2].

The local outlier factor, LOF, proposed in [3], is a well-known density-based algorithm for the detection of local outliers in static data. LOF measures the local deviation of data points with respect to their *K* nearest neighbors, where *K* is a user-defined parameter. This kind of method can be useful in several applications, such as detecting fraudulent transactions, intrusion detection, direct marketing, and medical diagnostics. Later, the concept of LOF was extended for incremental databases [4], and for streaming environments [5,6]. However, recent advances in LOF-based outlier detection algorithms for data streams, MILOF [5] and DILOF [6], do not consider variations in data that may change over time. For example, there may appear a new cluster of data points over time in the data streams. In addition, algorithms for data streams need to avoid using outdated data. To handle the data streams, the algorithms utilize a fixed window size to limit the number of data points held in memory by summarizing previous data points. These recent studies base their summaries only on the distribution of previous data; i.e., they do not take the sequence of data into account. The fact that these methods lack a mechanism for the removal of outdated data can greatly hinder their performances. Imagine a situation where sensors installed near a factory are used to detect the emission of PM2.5 pollutants. If pollutants were emitted on more than one occasion (with an intermittent period of normal concentrations), then the fact that the initial pollution event is held in memory might prevent the detection of subsequent violations. In other words, if the previous pollution event is held in memory for longer time, the next pollution event will be treated as an inlier and the method could not detect next pollution event.

Moreover, limited memory and computing power impose limitations on window size and thus on model performance because limitations on memory capacity and computational power necessitate the elimination of some previous data points. However, setting an excessively small window size can degrade performance because we can only hold a few data points in memory and hence there may be lack of neighboring data points with similar features, which affects the outlier scores.

A data stream potentially contains an infinite number of data points: $S=\left\{s_{1},s_{2},\ldots ,s_{t},\ldots \right\}$. Each data point $S_{t}\in R^{D}$ is collected at time *t*. We need to consider the following constrains for applications in data stream environments.

- Continuous data points (usually infinite).
- Limited memory and limited computing power.
- Real time responses for processed data.

Our goal is to detect outliers by calculating the LOF score for each data point. In addition, we are focusing on detecting outliers in data stream. Therefore, the following constraints must be considered in the detection of outliers in a data stream.

- Memory limitations constrain the amount of data that can be held in memory. We need to consider this for handling unbounded data stream environment.
- The state of the current data point as an outlier/inlier must be established before dealing with subsequent data points. Note that we do not have any information related to subsequent data points appearing in the data stream.
- Adding new data may induce new clusters.
- Limited computing power needs to be utilized before new data arrives in the data stream. Therefore, the algorithms need to be efficient in terms of execution time.

In this study, we sought to resolve these issues by developing a (1) time-aware and density-summarizing incremental LOF (TADILOF) and (2) a method to approximate the value of LOF. For time-aware summarization, we include a time component, also termed time indicator, with each data point. The inclusion of a time component in the summary phase makes it possible to consider the sequential order of the data, and thereby deal with concept drift and enable the removal of outdated data points. Basically, every data point is assigned a time indicator referring to the point at which it was added to the streaming data. When a new data point arrives, the time indicators of K-nearest neighbor data points are updated if the newly added data point is not judged as an outlier. Using this strategy, the data points near to new data points are updated with the current time indicator and therefore these data points are less likely to be removed in the summarization phase. Thus, our proposed method is more likely to follow the variations in data that may change over time.

Furthermore, we propose a method to calculate approximate LOF score based on the summary information of previous data points. Note that this involves estimating the distances between newly-added data points and potential deleted neighbors (i.e., data points deleted in a previous summary phase). In the proposed method, LOF score is used to decide whether a newly added data point is an outlier or not in accordance with a LOF threshold. LOF score represents the outlierness of the data points based on the local densities defined using K-nearest neighbor data points. In addition, LOF score is able to adjust for the variations in the different local densities [2]. If the newly added data point is detected as an outlier as per LOF threshold, we use a second check based on proposed approximate LOF score to finally decide whether it is an outlier or not.

To maintain the data in the window, we use the concept of a landmark window strategy as used in the recent studies, MILOF [5] and DILOF [6], for local outlier detection in data streams. When the window is filled with the data points, we summarize the window to make space available for new data points by removing the old and less important data identified by the proposed summarization method. In our proposed summarization method, we summarize the data points of complete window using three quarters of the window. Then, one quarter size of window becomes available for new data points. We discuss the details of our summarization method in Section 3.2.

To limit the data to fit into available memory, the sliding window technique used in several applications for data streams is also an option. In the sliding window technique, all the old data points are deleted that cannot fit into memory. However, this may degrade the performance of local outlier detection because new events cannot be differentiated from some past events, and the accuracy of the estimated local outlier factor of data points will be affected if the histories of earlier data points are deleted [5]. Therefore, we use the landmark window strategy. In addition, the proposed strategies of using a time indicator and approximate LOF are suitable in combination with a landmark window for local outlier detection accuracy.

In addition, to evaluate the performance of our proposed method, we executed extensive experiments against the state-of-the-art algorithms on various real datasets. The results of experiments illustrate that the proposed algorithm outperforms state-of-the-art competitors in terms of AUC while achieving similar performance in terms of execution time. The results of experiments validate the effectiveness of the proposed method to use the time component and approximate LOF, which help to achieve better AUC.

Moreover, we applied the proposed method to a real-world data streaming environment for the monitoring of the air quality. The Taiwanese monitoring system referred to as the location-aware sensing system (LASS) employs 2000 sensors, each of which can be viewed as an individual data stream. We used the proposed system to detect outliers in each of these data streams. We call this type of outlier a temporal outlier because such outliers are compared with historical data points from the same device. We then combine the position of every device to facilitate the detection of spatial outliers and pollution events based on outliers from the neighboring devices.

The main contributions of this work are as follows.

- We developed a novel algorithm to detect outliers in data streams. The proposed approach is capable of adapting the changes in variations of data over time.
- We developed an algorithm to calculate approximate LOF score in order to improve model performance.
- Extensive experiments using real-world datasets were performed to compare the performance of the proposed scheme with those of various state-of-the-art methods.
- The efficacy of the proposed scheme was demonstrated in a real-world pollution detection system using PM2.5 sensors.

The rest of this paper is organized as follows. In Section 2, we discuss related works. Then, we introduce the proposed method in Section 3. In Section 4, we describe our experiments and a performance evaluation of the proposed method. Section 5 demonstrates a case study based on our proposed method for monitoring air quality and detection of pollution events. Finally, conclusions are presented in Section 6.

## 2. Background and Related Work

Outlier and anomaly detection on large datasets and data streams is a very important research area that has been useful for several applications [1,2,7]. Some studies focus on detecting global outliers, whereas other studies focus on detecting local outliers [1,2]. Different approaches have been studied for outlier detection, such as distance-based methods, density-based methods, and neural network-based methods [8].

In addition, clustering techniques can also be used for outlier detection. Therefore, we discuss some works on clustering and outlier detection based on clustering. In [9], the authors discussed a method for incremental K-means clustering. In the incremental database, this approach is better than traditional K-means. Similarly, the study in [10] proposes IKSC, incremental kernel spectral clustering, for online clustering in dynamic data. Another study in [11] discusses various machine learning approaches for real-world SHM (structural health monitoring) applications. The authors discuss the temporal variations of operational and environmental factors and their influences on the damage detection process. In [12], the authors propose enhancement of density-based clustering and outlier detection based on clustering. In addition, the authors discuss the approach for parameter reduction for density-based clustering. In [13], the authors propose a density-based outlier detection method using DBSCAN. First, the authors compute the minimum radius of an accepted cluster; then a revised version process of DBSCAN is used to further fit for data clustering and the decision of whether each point is normal or abnormal can be made. In [14], the authors provide survey of unsupervised machine learning algorithms that are proposed for outlier detection. In [15], the authors propose a cervical cancer prediction model (CCPM) for early prediction of cervical cancer using risk factors as inputs. The authors utilize several machine learning approaches and outlier detection for different preprocessing tasks.

The local outlier factor (LOF) [3] is a well-known density-based algorithm for the detection of local outliers in static data. This method can be useful in several applications, such as detecting fraudulent transactions, intrusion detection, direct marketing, and medical diagnostics [16,17,18]. Based on LOF, the study in [19] proposed a method to mine top-n local outliers. Later, the concept of LOF was extended for dynamic data—for instance, incremental LOF (iLOF) [4] was made for incremental databases, and MiLOF [5] and DILOF [6] were made for streaming environments. The application of LOF to incremental databases requires updating every previous data point and the recalculation of the LOF score, both of which are computationally intensive. iLOF reduces the time complexity to O(nlogn) by updating the LOF score of data points affected by newly-added data points. Unfortunately, this approach is inapplicable to data streams with limited memory resources. MiLOF leverages the concept of K-means [20] to facilitate outlier detection in data streams by overcoming the space complexity of iLOF (i.e., $O(n^{2})$). MiLOF uses a fixed window size to limit the number of data points held in memory by summarizing previous data points through the formation of K-cluster centers. Note, however, that MiLOF is prone to the loss of density information and a large number of points are required to represent sparse clusters. DILOF was developed to improve the summarization process using the nonparametric Rényi divergence estimator [21] to select minimum divergence subset from previous data points. However, neither MiLOF nor DILOF consider the concept-drift [22,23] of data in data streams to avoid using outdated data [24]. Furthermore, MiLOF and DILOF base their summaries only on the distribution of previous data; i.e., they do not take the sequence of data into account.

Some other methods based on LOF have been proposed for top-n outlier detection. In [25], the authors proposed the TLOF algorithm for scalable top-n local outlier detection. The authors proposed a multi-granularity pruning strategy to quickly prune search space by eliminating candidates without computing their exact LOF scores. In addition, the authors designed a density-aware indexing mechanism that helps the proposed pruning strategy and the KNN search. In [26], the authors proposed local outlier semantics to detect local outliers by leveraging kernel density estimation (KDE). The authors proposed a KDE-based algorithm, KELOS, for top-n local outliers over data streams. In [27], the authors proposed the UKOF algorithm for top-n local outlier detection based on KDE over large-scale high-volume data streams. The authors defined a KDE-based outlier factor (KOF) to measure the local outlierness score, and also proposed the upper bounds of the KOF and an upper-bound-based pruning strategy to reduce the search space. In addition, the authors proposed LUKOF by applying the lazy update method for bulk updates in high-speed large-scale data streams.

Since this study proposes a method to find local outliers in data streams, we discuss LOF, iLOF, MiLOF, and DILOF in the following subsections.

### 2.1. LOF and iLOF

LOF scores are computed for all data points according to parameter K (i.e., the number of nearest neighbors). The LOF score is calculated as follows:

**Definition** **1.** d(p,o) is the Euclidean distance between two data points p and o.

**Definition** **2.** K-distance(p), $d_{K}\left(p\right)$, is defined as the distance between data point p and its $K^{th}$ nearest neighbor.

**Definition** **3.** *Given two data points p and o, reachability distance $\mathrm{reach-dis}t_{K}\left(p,o\right)$ is defined as:*

(1) $$\mathrm{reach-dis}t_{K}\left(p,o\right)=max\left\{d\left(p,o\right),\mathrm{K-distance}\left(o\right)\right\}$$

**Definition** **4.** *Local reachability density of data point p, LRD(p), is derived as follows:*

(2) $$LRD\left(p\right)={\left(\frac{1}{K}*\sum_{o\in N_{K}\left(p\right)}\mathrm{reach-dist}\left(p,o\right)\right)}^{-1}$$

*where $N_{K}$ is the set of K nearest neighboring data points of point p, and K is a user-defined parameter.*

**Definition** **5.** *Local outlier factor of data point p, LOF(p), is obtained as follows:*

(3) $$LOF\left(p\right)=\frac{1}{K}*\sum_{o\in N_{K}\left(p\right)}\frac{LRD_{K}\left(o\right)}{LRD_{K}\left(p\right)}$$

If the LOF score of a data point is greater than or equal to the threshold, then that data point is considered an outlier.

LOF is used to calculate the LOF scores only once. iLOF was developed to deal with the problem of data insertion, wherein we update only the previous data points that are affected by the new data point. Note that iLOF is not applicable to the detection of outliers in streaming data, due to the fact that there is no mechanism for the removal of outdated points. In addition, real-world applications lack the memory resources required to deal with the enormous (potentially infinite) number of data points generated by streaming applications.

Since LOF and iLOF are not suitable for data streams, MiLOF [5] was proposed for the detection of outliers in streaming data. We discuss MiLOF in the next subsection.

### 2.2. MiLOF

MiLOF [5] was developed for the detection of outliers in streaming data using limited memory resources. Essentially, MiLOF overcomes the memory issue by summarizing previous data points. MiLOF is implemented in three phases: insertion, summarization, and merging. Note that the insertion step of MiLOF is similar to that of iLOF. When the number of points held in memory reaches the limit imposed by window size *b*, the summarization step is invoked, wherein the K-means algorithm is used to find *c* cluster centers to represent the first $\frac{b}{2}$ data points, after which the insertion step is repeated iteratively. In the merging phase, weights are assigned to each cluster center based on the number of associated data points. The weighted K-means algorithm is then used to merge the new cluster center with the old cluster center. When using MiLOF, the total amount of data held in memory does not exceed m=b+c. MiLOF can be used to reduce memory and computation requirements; however, it does not preserve the density of the original dataset within the summary, which is crucial to detection accuracy.

### 2.3. DILOF

Being similar to MILOF, DILOF is a density-based local outlier detection algorithm for data streams that utilizes LOF score to detect outliers. DILOF is implemented in two phases: detection and summarization. The detection phase, which is called last outlier-aware detection (LOD), uses the iLOF technique to calculate LOF values when new data points are added to the dataset. DILOF then classifies the data points within the normal class or as an outlier. The summarization phase, which is called nonparametric density summarization (NDS), is activated when the number of data points reaches the limit defined by window size *W*. DILOF uses the nonparametric Rényi divergence estimator [21] to characterize the divergence between the original data and summary candidate. The gradient descent method is then used to determine the best summary combination. Summarization compiles half of the data $X=\left\{x_{1},x_{2},\ldots ,x_{W/2}\right\}$ within a space one quarter the size of the window size $Z=\left\{z_{1},z_{2},\ldots ,z_{W/4}\right\}$ by minimizing the loss function. There are four terms in the loss function. In the following, we introduce them one by one.

The first term is the Rényi diversity between the summary candidate and the original data. Renyi diversity is calculated using Equation (4), as follows:

(4) $$\sum_{n=1}^{W/2}y_{n}\frac{p_{K}\left(x_{n}\right)}{v_{K}\left(x_{n}\right)}$$

In Equation (4), $y_{n}$ is the binary decision variable of each data point $x_{n}$. Data point $x_{n}$ is selected when $y_{n}$ equals 1 and discarded when $y_{n}$ equals 0. However, assessing every subset combination to determine the minimum loss values is impractical. NDS resolves this issue by relaxing the decision variable to produce an unconstrained optimization problem, where $y_{n}$ becomes a continuous variable. Using the gradient descent method, NDS selects the best combination of $x_{n}$—i.e., the half of parameter set $y_{n}$ with the highest values. $p_{k}\left(x_{n}\right)$ is the Euclidean distance between data point $x_{n}$ and its *K*th-nearest neighbor in X. $v_{k}\left(z_{n}\right)$ is the Euclidean distance between data point $z_{n}$ and its *K*th-nearest neighbor in Z. This term is given by the Rényi divergence estimator.

The second term is the shape term, which preserves the shape of the data distribution by selecting data points at the boundary of clusters, such that the data point within the boundary always has a higher LOF value. This term is shown as Equation (5).

(5) $$-\sum_{n=1}^{W/2}y_{n}e^{LOF_{K}\left(x_{n}\right)}$$

The third and fourth terms are regularization terms. The third term is used to control $y_{n}$ close to 0–1. It is important to avoid excessively high $x_{n}$ values, which would render other data points ineffective. The fourth term is used to select half of all data points. These terms are shown in Equation (6).

(6) $$\sum_{n=1}^{W/2}\psi_{0,1}\left(y_{n}\right)+\frac{\lambda }{2}{\left(\sum_{n=1}^{W/2}y_{n}-\frac{W}{4}\right)}^{2}$$

Combining all of the components, we obtain the loss function of DILOF as follows:

(7) $$\begin{array}{c} \min_{y}\sum_{n=1}^{W/2}y_{n}\frac{p_{k}\left(x_{n}\right)}{v_{k}\left(x_{n}\right)}-\sum_{n=1}^{W/2}y_{n}e^{LOF_{k}\left(x_{n}\right)}\\ +\sum_{n=1}^{W/2}\psi_{0,1}\left(y_{n}\right)+\frac{\lambda }{2}{\left(\sum_{n=1}^{W/2}y_{n}-\frac{W}{4}\right)}^{2} \end{array}$$

The gradient descent method is then used to obtain the optimal result as shown in Equation (8).

(8) $$\begin{array}{c} {y_{n}}^{\left(i+1\right)}={y_{n}}^{\left(i\right)}-\eta \left\{\sum_{x\in C_{K,n}}\frac{p_{K}\left(x\right)}{v_{K}\left(x\right)}+\frac{p_{K}\left(x_{n}\right)}{v_{K}\left(x_{n}\right)}-e^{LOF_{K}\left(x_{n}\right)}\right.\\ +\left.{\psi_{0,1}}^{{}^{\prime }}\left({y_{n}}^{i}\right)+\lambda \left(\sum_{n=1}^{W/2}{y_{n}}^{\left(i\right)}-\frac{W}{4}\right)\right\} \end{array}$$

In Equation (8), ψ is the learning rate, i is the number of iteration, and $C_{\left(K,n\right)}$ is a set of data points that have $x_{n}$ as their *K*th-nearest neighbor in Z. Interested readers are referred to the DILOF paper [6] for details on the calculation of $C_{\left(k,n\right)}$. After the decision variable has been updated, the larger half is selected as the summary point. Following this summarization phase, half of all data points are summarized into a quarter of all data points. This leaves a space equal to one quarter of the window size into which new data points can be inserted.

The DILOF method lacks a mechanism by which to remove outdated data or compensate for concept drift. NDS calculates only the difference in density in selection of a summary point. We therefore added the concept of time to differentiate outdated data points.

## 3. Proposed Method: TADILOF

In this section, we outline the proposed TADILOF algorithm and approximate LOF score. Algorithm 1 presents the pseudocode of the TADILOF algorithm. Our scheme also uses density to select the summary; therefore, we have two phases: detection and summarization. In the detection phase, we include a step in which previous information is used to obtain the approximate LOF, which is then used to determine whether the newly-added point is an outlier. This detection phase is referred to as ODA, outlier detection using approximate LOF. We add a time component to the summarization phase, and therefore refer to it as time-aware density summarization (TADS). We provide the details of procedures TADS and ODA in the following subsections. The approximate LOF score is calculated only when there is information from previous data points. Therefore, we introduce the time component before obtaining the approximate LOF score.
**Algorithm 1** TADILOF algorithm**Input:** 
DS: A data stream $D=\{d_{1},d_{2},\ldots ,d_{t},\ldots \}$,
  Window size: *W*,
  Number of neighbor: *K*,
  Threshold: θ,
  Step size: η,
  Regularization constant: λ,
  Maximum number of iteration: *I*
**Output:** The set of outliers in streams
 1: dataInMemory={};
 2: outlierSet={};
 3: **while** a new data point $d_{t}$ is in stream **do**
 4:      dataInMemory.add($d_{t}$)
 5:      $LOF_{k}\left(d_{t}\right)$ = ODA($d_{t}$,outlierSet,θ)
 6:      **if**
$LOF_{k}\left(d_{t}\right)>\theta$
**then**
 7:          outlierSet.add($d_{t}$)
 8:      **if**
dataInMemory.length >W
**then**
 9:          dataInMemory=TADS(dataInMemory,η,λ,*I*)
10: **end while**


### 3.1. Time Component

Addition of a time component to this type of task allows the model to distinguish old data from new, thereby making it possible to recognize concept drift over time. For example, daytime readings might not be explicitly differentiated from nighttime readings in the PM2.5 data, despite the fact that time of day plays an important role in PM2.5 concentrations. Another example is the degree to which purchasing behavior varies over time as a function of the strength of the economy. The addition of a time component also provides a mechanism by which to remove outdated data, which might otherwise compromise model performance.

In this study, we include a time component in the summarization phase. Basically, every data point is assigned a time indicator $t_{i}$ referring to the point at which it was added to the streaming data. In other words, the time indicators describe the age of every data point. The difference between $t_{i}$ and the current time point corresponds to the length of time that data point $d_{i}$ has existed in the dataset. The objective is to discard outdated data and preserve newer data points, which are presumed to more closely approximate the current situation. TADILOF refreshes data points close to the current data point and updates the time indicator of points neighboring the new data point, as shown in the following equation. Fortunately, this does not incur additional calculations due to the fact that we have already identified the neighbors of the new data in the LOF process.

(9) $$t_{i}=t_{new},\;if\;d_{i}\in N_{K}\left(d_{new}\right)$$

Refreshing the time indicator of each data point enables our loss function to select data points that fit the current concept. Thus, a new model can be used to select data points in accordance with the density as well as the concept(s) represented by the current data streams. When TADS is triggered to summarize previous data points, it calculates the time difference t_diff between summarized time stamp $t_{s}$ and the time stamp of data point $d_{i}$ as follows:

(10) $$t\_diff_{i}=max\left\{t_{s}-t_{i}-\alpha *W,0\right\}$$

In Equation (10), α is a hyperparameter indicating the amount of time that must elapse before TADILOF designates data as outdated and removes them. For example, $\alpha =\frac{W}{4}$ means that any data point with a time difference of less than one quarter of the window size is less likely to be selected for removal by the objective function. We present TADS in the next subsection.

### 3.2. Time-Aware Density Summarization (TADS)

**Figure 1:**
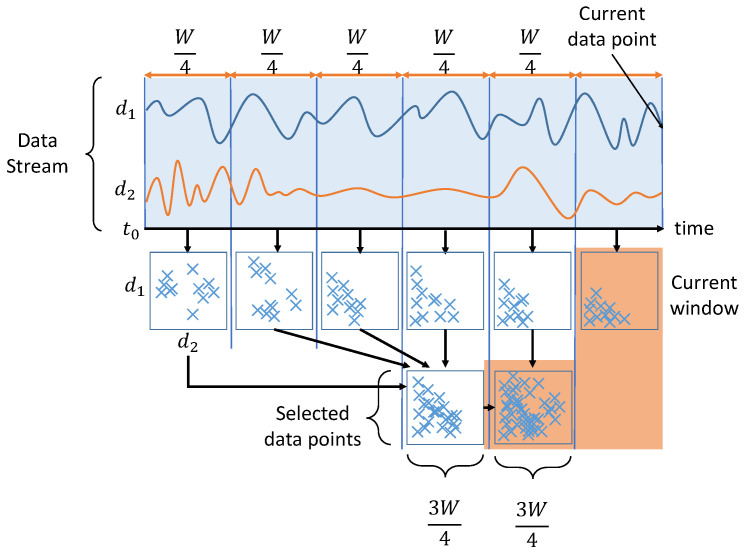
The summarization phase of TADILOF.

Figure 1 presents the proposed TADS (in the TADILOF algorithm), which differs from NDS (in the DILOF algorithm). Note that NDS always retains the most recent half window of data points and summarizes the older half within a quarter size window. By contrast, TADS summarizes data points from three quarters of the window, and does not necessarily retain only the latest data. Rather, the TADS mechanism considers the density and the age of the data points. The time term is added to the TADS loss function as follows:

(11) $$\begin{array}{c} \min_{y}\sum_{n=1}^{W}y_{n}*t\_diff_{n}+\sum_{n=1}^{W}y_{n}\frac{p_{K}\left(x_{n}\right)}{v_{K}\left(x_{n}\right)}-\sum_{n=1}^{W}y_{n}e^{LOF_{K}\left(x_{n}\right)}\\ +\sum_{n=1}^{W}\psi_{0,1}\left(y_{n}\right)+\frac{\lambda }{2}{\left(\sum_{n=1}^{W}y_{n}-\frac{3W}{4}\right)}^{2} \end{array}$$

The details of the TADS procedure are shown in Algorithm 2.
**Algorithm 2** Procedure TADS**Input:** set of data point in memory $X=\{x_{1},x_{2},\ldots x_{W}\}$,
  Window size: *W*,
  Step size: η,
  Regularization constant: λ,
  Maximum number of iteration: *I*
**Output:** summary set
 1: **for each**
datapointx∈X
**do**
 2:      **if**
$LOF_{k}\left(x\right)<historicalLOF\left(x\right)$
**then**
 3:          update LOF,LRD and meanDistance
 4: **end for**
 5: $Y=\{y_{1},y_{2},\ldots y_{W}\}$
 6: **for each** 
decisionvariablesy∈Y
**do**
 7:      *y* = 0.75
 8: **end for**
 9: **for**
*i* = 1:*I*
**do**
10:      η=η∗0.95
11:      **for**
*n* = 1:*W*
**do**
    ▹ Using objective function, calculate the score of each data point for selection in the summary set.
12:      ${y_{n}}^{\left(i+1\right)}={y_{n}}^{\left(i\right)}-\eta \left\{t\_diff_{n}+\sum_{x\in C_{K,n}}\frac{p_{k}\left(x\right)}{v_{k}\left(x\right)}-e^{LOF_{k}\left(x_{n}\right)}{\psi_{0,1}}^{{}^{\prime }}\left({y_{n}}^{i}\right)+\lambda \left(\sum_{n=1}^{W}{y_{n}}^{\left(i\right)}-\frac{3W}{4}\right)\right\}$
13:      **end for**
14: **end for**
15: Project Y into binary domain
16: **for** n=1:$\frac{3W}{4}$
**do**
17: 
    $Z\leftarrow Z\cup \{x_{n}\}$
18: **end for**
19: Return *Z*


### 3.3. LOF Score and ODA (Outlier Detection Using Approximate LOF)

**Figure 2:**
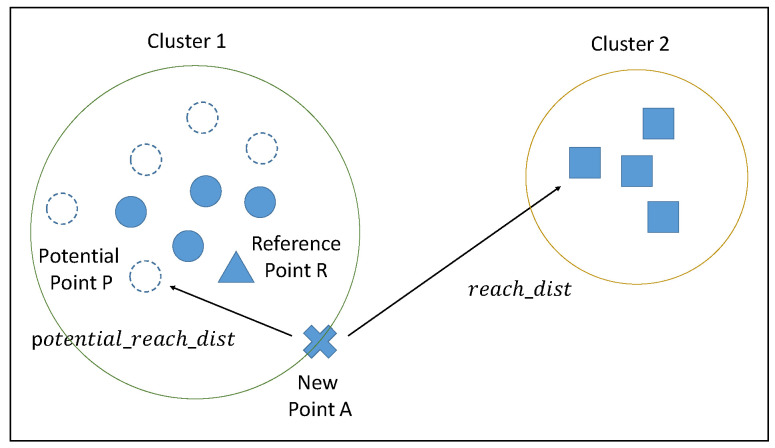
The case of new point to calculate LOF score with point from other cluster.

Limitations on memory capacity and computational power necessitate the elimination of some previous data points; however, setting an excessively small window size can degrade performance. Let us take an example shown in Figure 2 with two local clusters from the data stream. The symbols in different shape do not represent different kind of data points in a data stream. We have just make different symbols to represent two different local clusters of data points from data stream in Figure 2. In the example in Figure 2, new point A sits very close to cluster 1, but some of the points in that cluster were deleted in the previous summarization phase, with the result that the new point is unable to find a sufficient number of neighbors in cluster 1. This means that LOF must be calculated using points from cluster 2, which could present the new point as an outlier. We sought to overcome this issue by calculating approximate LOF scores, which are then saved with the LRD and the mean distance between each point to neighbors in every summarization phase. This saved information can then be used to calculate the reachability of potential neighbors.

Assume that new point *A* is added to the dataset. If the calculated LOF exceeds the threshold, then the algorithm classifies it as an outlier. At the same time, historical information related to reference point *R* (a KNN neighbor of *A*) is used to find potential neighbor point *P* as a function of historical distance between *R* and its neighbors. Following the identification of the reference point *R* and its potential neighbor point *P*, the approximate LOF value is calculated to reassess whether the data point in question should be classified as an outlier or an inlier.

Calculation of the approximate LOF score requires preservation of some of the information in the previous window. In the summarization phase, the LOF score of any data point selected for inclusion in the first summary is retained as its historical LOF score. Note that its historical LRD and the mean distance to its neighbors are also preserved. For any data point selected for the initial and subsequent summarization, we compare the current LOF score with its historical LOF score. In cases where the current LOF score is lower, the associated information is updated. Note that a lower LOF score is indicative of the density typical of inliers.

Point A has K-nearest neighbors. Our aim is to identify the neighbor with the lowest product of historical LOF score and Euclidean distance between A and itself. That neighbor is then used as a reference point R by which to calculate the approximate LOF score of A.

We can use the historical LRD of R to obtain the mean reachability distance between R and P using the following equation:

(12) $$\mathrm{mean-reach-dist}\left(R,P\right)=\frac{1}{historicalLRD(R)}$$

Our objective is to identify potential neighbors of new point A. Even though the current state indicates that A is an outlier, it may in fact be an inlier if some of its neighbors avoided deletion in the previous few windows.

There are three scenarios in which new point *A*, reference point *R*, and potential neighbor *P*, which represents a deleted data point, could be distributed in ODA. In Definition 1d(R,P) is used to represent the mean Euclidean distance between *R* and *P*. Using Definition 3, reach-dist(R,P) indicates the mean reachability distance between *R* and *P*. Before we discuss these three scenarios, it is necessary to discuss the distribution of potential neighbors. Potential neighbor *P* can be in any position, including the space between the reference point and the new point. It is infeasible to record all potential neighbor positions; therefore, we use the case where the potential neighbor is located at the greatest distance between the new data point and itself. We then use the mean distance between *R* and its historical neighbors and the mean reachability distance to calculate the approximate reachability distance between *A* and *P*.

**Figure 3:**
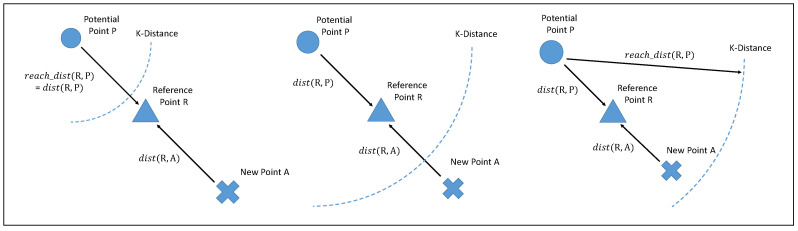
Three scenarios of a potential neighbor, a reference point, and a new point.

In the first scenario (Figure 3 left), reachability distance reach-dist(R,P) is equal to Euclidean distance d(R,P), which is larger than K-distance(P). In this scenario, ODA can use d(R,P)+d(R,A) to cast the mean approximate reachability distance between *A* and *P*. In the second scenario (Figure 3 middle), reach-dist(R,P) is larger than d(R,P) but less than d(R,P)+d(R,A). In this case, ODA can also use d(R,P)+d(R,A) to cast the mean approximate reachability distance between *A* and *P*. In the third scenario (Figure 3 right), reach-dist(R,P) is larger than d(R,P)+d(R,A). In this case, ODA can use reach-dist(R,P) to represent the mean approximate reachability distance reach-dist(A,P).

By assembling these, we can obtain the approximate mean reachability distance between point *P* and *A* using the following equation:

(13) $$\begin{array}{c} \mathrm{mean-reach-dist}(A,P)=\\ max\left\{d\left(R,P\right)+d\left(R,A\right),\;\frac{1}{historicalLRD(R)}\right\} \end{array}$$

After obtaining the approximate mean reachability distance of point *A*, we can calculate the approximate LRD of *A* using Equation (2) (Definition 4), based on the fact that LRD is the reciprocal of the mean reachability distance.

(14) $$ApproximateLRD\left(A\right)=\mathrm{mean-reach-dist}{\left(A,P\right)}^{-1}$$

ODA then calculates the sum of LRD of *P* using Definition 5, as follows:

(15) mean-LRD(P)=historicalLOF(R)∗historicalLRD(R)

The approximate reachability distance and average LRD of the potential neighbor are then used to compute the approximate LOF using Definition 5, as follows:

(16) $$ApproximateLOF\left(A\right)=\frac{\mathrm{mean-LRD}(P)}{ApproximateLRD(A)}$$

ODA can use this approximate LOF to determine whether A is an outlier or an inlier. The pseudocode of the ODA procedure is shown in Algorithm 3.
**Algorithm 3** Procedure of ODA**Input:**data point $x_{t}$
  set of data point in memory $X=\{x_{1},x_{2},\ldots x_{t}\}$,
  threshold: θ,
  set of detected outlier: outlierSet
**Output:** LOF score of $x_{t}$
 1: Using incremental LOF technique updates all reverse KNNs of $x_{t}$
 2: $N_{K}\left(x\right)$ = All KNNs of $x_{t}$
 3: **for each** 
neighbor
$n\in N_{K}\left(x\right)$
**do**
 4:      updating time stamp of *n*
 5: **end for**
 6: Compute $LOF_{k}\left(x_{t}\right)$
 7: **if** 
$LOF_{k}\left(x_{t}\right)>\theta$
**then**
 8:      Reference Point R= ${arg min}_{r\in N_{K}A}historicalLOF\left(r\right)*d\left(r,A\right)$
 9:      Find the approximate reachability distance using Equation (12)
10:      Find the approximate LRD of $\left(x_{t}\right)$ using Equation (13)
11:      Use historical LRD of R and historical LOF of R to find mean of LRD of potential neighbors by Equation (14)
12:      Find the approximate LOF of $\left(x_{t}\right)$ using Equation (15)
13:      **if** approximate LOF of $(x_{t})>$ Threshold **then**
14:          outlierSet.add($x_{t}$)


### 3.4. Time and Space Complexity

In DILOF [6], the authors analyzed time complexity from the perspectives of summarization and detection separately. Note that time complexity of DILOF in the detection phase is O(W), whereas time complexity of DILOF in the summarization phase is $O({\frac{W}{2}}^{2})$. The space complexity of DILOF algorithms is O(W∗D), where *D* is the dimensionality of the data points.

In the following, we discuss the detection phase of the proposed algorithm, TADILOF, in which we calculate the approximate value of the points classified as outliers by the LOF score. Let us assume that *z* is the number of points that are classified as outliers. In our proposed detection phase, O(K) is incurred in calculating the approximate LOF score for each point. Thus, O(W+z∗K) indicates the time complexity in the detection phase. However, the number of neighbors *K* is far less than window size *W*. Therefore, the cost incurred in the detection phase is O(W).

The time complexity of TADILOF in the summarization phase is $O(W^{2})$. TADILOF tends to require more time than DILOF. However, the execution times in the experiments were still very close.

The additional space complexity associated with the proposed method includes the time indicator, historical LOF, historical LRD, and mean neighbor distance. Note that the size of the data in the summary is $\frac{3W}{4}$. Therefore, the total cost is O(3W). From this, we can see that the space complexity of TADILOF with approximate LOF is O(W∗(D+3)).

## 4. Performance Evaluation

In this section, we compare the performance of TADILOF with the state-of-the-art, DILOF [6] and MiLOF [5] algorithms. In addition, we have included results of experiments from iLOF [4] algorithm on some datasets. We downloaded the implementation of DILOF and iLOF from URL provided in [6]. In [6], two versions of DILOF were implemented. One without “skipping scheme” and another with “skipping scheme”. We discuss the *skipping scheme* and the related experiments in Section 4.4. First, we describe the datasets and experiment settings, i.e., the parameters used in the experiments. We then examine the performance of each algorithm.

### 4.1. Datasets

The performance of the proposed method was evaluated by applying it to various datasets, which are shown in Table 1. We downloaded these preprocessed datasets from ODDS, Outlier Detection Datasets, Library [28]. These datasets were originally from UCI Machine Learning Repository (https://archive.ics.uci.edu/ml/index.php). ODDS Library provides preprocessed versions of these datasets. For the details about these datasets and information on preprocessing, we refer the readers to the ODDS Library website (http://odds.cs.stonybrook.edu/).

### 4.2. Experiment Settings

The same set of hyperparameters were used for TADILOF and DILOF. The learning rate and maximum number of gradient descent iterations were set at 0.3 and 0.001, respectively. The K-nearest neighbors were 8 for all of the datasets. These parameters were suggested in DILOF [6] and we have used the same parameters in our experiments for comparisons to other algorithms. In addition, we ran another experiment for different *K* values. Some of the preprocessed datasets contained all the outliers grouped together (as a class) at the beginning or end. Some datasets had outliers scattered among inliers. We therefore shuffled datasets of the former kind before running the algorithms. The last column in Table 1 shows whether we shuffled the dataset or not, where “true” means we shuffled the dataset. We also assessed model performance using windows of various sizes, due to the importance of this parameter in terms of memory usage and computation time. For small datasets, we selected a small window size W={100,120,140,160,180,200}. Similarly, for larger datasets, we selected larger window size W={100,200,300,400,500,600,700}. For LOF score thresholds, we use LOF_Thresholds={0.1,1.0,1.1,1.15,1.2,1.3,1.4,1.6,2.0,3.0} which were used in DILOF implementation. The same thresholds were used in the experiments, and false positive rate (FPR) and true positive rate (TPR) were calculated for each threshold. Then AUC in ROC space was calculated for all the algorithms. All experiments were performed on a PC with Intel Core i7-3770 3.4 GHz, 32 GB RAM, and Windows 10 64-bit operating system. The algorithms were implemented in C++ programming language.

### 4.3. Experimental Results

#### 4.3.1. AUC, Execution Time, and Memory Usage

We evaluated MiLOF, DILOF, and TADILOF in terms of AUC and execution time on various datasets. As reported in [6], “DILOF without skipping scheme” had better performance than “DILOF with skipping scheme” in the datasets except for “HTTP KDD Cup 99” dataset. Therefore, we compare “DILOF without skipping scheme” with the proposed TADILOF in this section. We discuss the skipping scheme and related experiments on “HTTP KDD Cup 99” dataset in Section 4.4.

**Figure 4:**
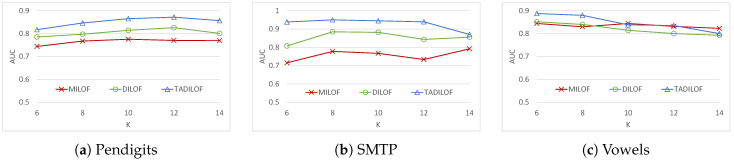
AUC on various datasets for different *K* values.

**Figure 5:**
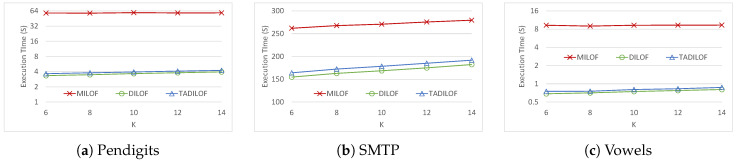
Execution time on various datasets for different *K* values.

First we ran experiments on Pendigits, SMTP, and Vowels datasets to assess the results for different *K* values. The window size was set at 140 for Pendigits and Vowels dataset while the window size was set at 400 for SMTP dataset. Figure 4 and Figure 5 show the results of this experiments, i.e., AUCs and execution times of MILOF, DILOF, and TADILOF algorithms. For the remaining experiments, we set *K* at 8, which was also used in DILOF [6].

**Figure 6:**
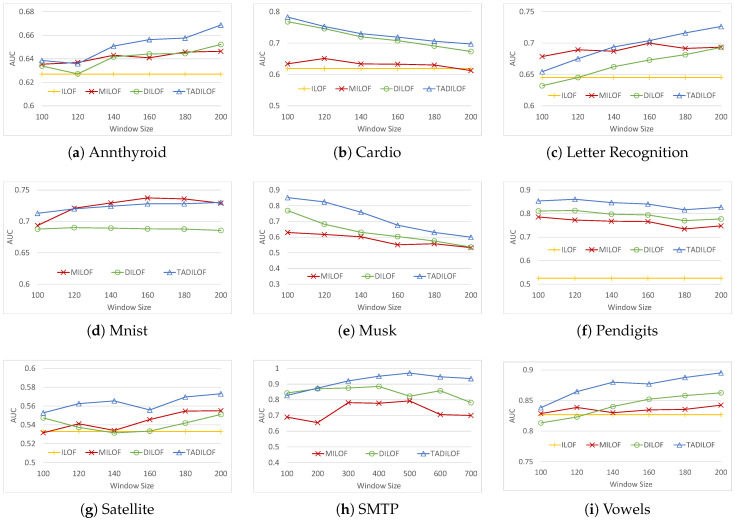
AUC on various datasets with different window sizes and K=8.

**Figure 7:**
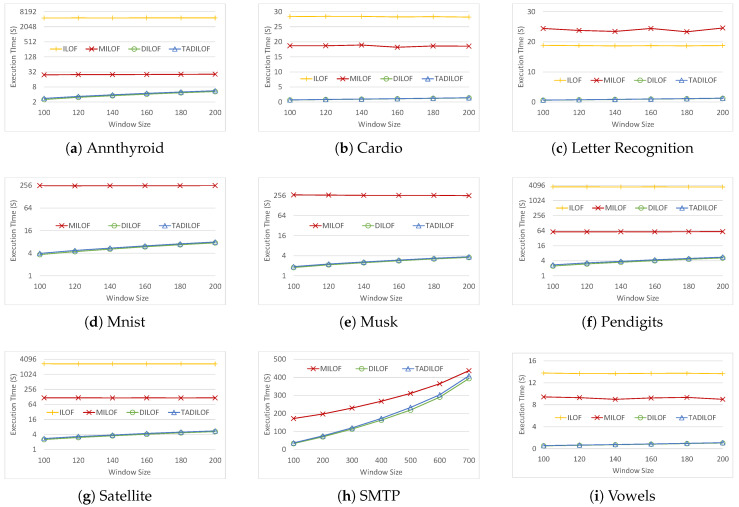
Execution time on various datasets with different window sizes and K=8.

Next, we ran the experiments on various datasets to assess the performances of the algorithms for different window sizes. Figure 6 and Figure 7 show the AUCs and execution timse of all the algorithms respectively. We can see that TADILOF outperformed MiLOF and DILOF in terms of AUC in most of the cases on various datasets. Next we discuss each experiment one by one.

Figure 6 illustrates that the AUC increases with the increase of window size on the Annthyroid, Letter Recognition, Mnist, Satellite, SMTP, and Vowels datasets. Similarly, the AUC decreases with the increase of window size on Cardio, Musk, and Pendigits datasets. In both the cases, TADILOF outperforms the competitors in terms of AUC for most of the window sizes on all these datasets. In terms of AUC, TADILOF is a clear winner on Cardio, Musk, Pendigits, Satellite, and Vowels datasets.

On the Annthyroid dataset, both MiLOF and TADILOF have similar AUCs for window sizes 100 and 120. However, in the case of window sizes larger than or equal to 140, TADILOF outperforms all the competitors.

On the Letter Recognition dataset, TADILOF outperforms DILOF in terms of AUC. Similarly, MiLOF outperforms DILOF. In addition, MiLOF outperforms TADILOF in the case of window sizes smaller than 140. However, in the case of window sizes larger than 140, TADILOF outperforms MiLOF.

On the Mnist dataset, TADILOF has higher AUCs for some window sizes, whereas for other window sizes MiLOF has higher AUCs. Both MiLOF and TADILOF outperform DILOF in terms of AUC on Mnist dataset.

On the SMTP dataset with a relatively small window size (100, 200, and 300), the performances of TADILOF and DILOF were similar. However, for the window sizes larger than 300, TADILOF clearly outperformed DILOF in terms of AUC. When the window size exceeds 400, the performance of DILOF dropped dramatically due to its inability to remove outdated data. Increasing the window size beyond 500 led to a slight drop in AUC of TADILOF. However, TADILOF maintained AUC at above 0.9 for larger windows that exceeded window size 300.

The reasons behind the better performance of TADILOF are as follows. The method removes outdated data which might otherwise have influence on new data points, thereby preventing the identification of outliers. The ability to follow the concept drift of the data using time indicator was also shown to enhance performance. In addition, approximate LOF score calculated with the historical information provides the second chance to judge the data point as outlier or inlier. Using the time component for time-aware summarization helps one to eliminate too-old data from the summary. Thus, it prevents the influence of data which are too old. However, due to window size limitation, some not-so-old data may also be deleted. Thus storing some statistics for K-neighbors from previous window helps to judge the new data by applying second check based on approximate LOF if the new data point is detected as outlier based on current LOF score.

Figure 7 shows the performances of the algorithms in terms of execution time. Note that the y-axis is in log scale of base 2 in the figures for Annthyroid, Mnist, Musk, Pendigits, and Satellite datasets. Both DILOF and TADILOF significantly outperform iLOF and MiLOF in terms of execution time. Overall, the time complexity of TADILOF matched the values estimated in Section 3.4. The time consumption of TADS was similar to that of the original NDS. The only difference was the fact that TADS calculated the Rényi divergence between all data points in memory and three quarters of the data points. In contrast, NDS computed half of all data points and a quarter of all data points. The approximation of LOF values increased execution time only slightly. Nevertheless, TADILOF had a similar performance to DILOF in terms of execution time. Overall, the proposed algorithm outperformed state-of-the-art competitors in terms of AUC while achieving similar execution times.

**Figure 8:**
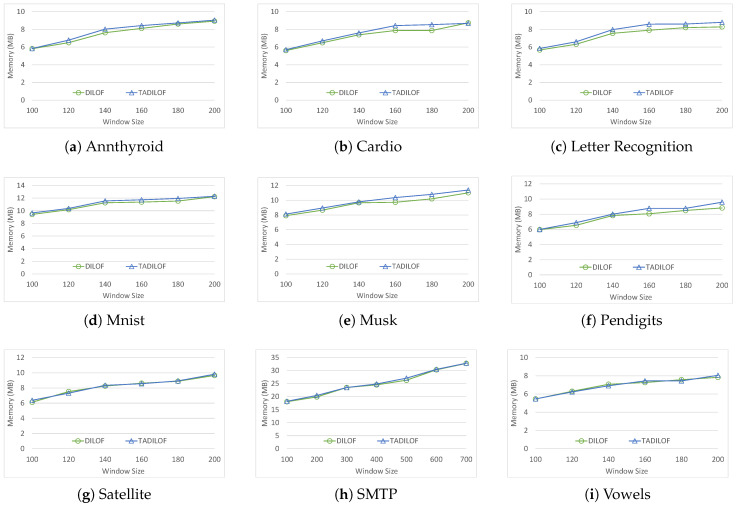
Memory usage on various datasets with different window sizes and K=8.

Similarly, Figure 8 shows the performances of DILOF and TADILOF on various datasets in terms of memory usage. We used Win32 API for reporting the memory usage of DILOF and TADILOF. Figure 8 demonstrates that in most of the cases, TADILOF used only a little more memory than DILOF. The results of experiments in terms of memory usage conformed with the theoretical analysis. Nevertheless, we can see from the results of experiments that both DILOF and TADILOF do not take much memory and are suitable for data stream environment.

#### 4.3.2. Precision, Recall, and F1 Score

On the same datasets, we investigated the precision, recall and F1 score for different window sizes and K=8. The Table 2, Table 3, Table 4, Table 5, Table 6, Table 7, Table 8, Table 9 and Table 10 show the precision, recall, and F1 score on various datasets for DILOF, TADILOF, and MILOF. In most cases, TADILOF had better precision and recall. Particularly, the recall values are much better than those of the other algorithms. Thus the F1 scores of TADILOF are the best. As for the precision, TADILOF performed better when the window size was larger.

### 4.4. Skipping Scheme for a Sequence of Outliers

In some cases, there may appear long sequence of outliers which can form a dense cluster of outliers. As reported in [6], in “HTTP KDD Cup 99” dataset there is a long sequence of outliers causing the algorithms to not perform well. In DILOF [6], the authors propose a skipping scheme to solve the sequence of outliers problem. Any point previously classified as an outlier point is set as the “last outlier,” before calculation of the Euclidean distance between the new point and the last outlier. If the Euclidean distance exceeds the average of all points to its first nearest neighbor, then that point is classified as an outlier and excluded from the database. Note however that the last outlier is identified using a particular threshold. Under these conditions, the fact that a different threshold could give a different last outlier means that it would be unreasonable to calculate AUC, considering that the likelihood of registering a true positive (TP) or false positive (FP) does not necessarily vary with the threshold. In this situation, the area under the curve is recalculated (i.e., the ROC is not continuous), such that AUC is unable to accurately indicate the performance of the model. Nonetheless, we propose to fix the threshold at a particular value to deal with this issue.

Note that the skipping scheme proposed with DILOF does not necessarily perform well on dense datasets, due to the fact that many points belonging to dense clusters might be skipped. For example, when there are a small number of sparse clusters in the memory, a new denser cluster appears. The distance between the points associated with this cluster will be larger than the average distance of previous data points, with the result that all of the points from this cluster are immediately discarded by the skipping scheme.

Thus, we modified the skipping scheme to calculate the average distance between new data points and their K neighbors. We then conducted a comparison of the distance between the last outlier and the new data point. In the event that the former is larger than the latter, then we immediately designate the new data point as an outlier and discard it. We implemented this modified skipping scheme with TADILOF.

**Figure 9:**
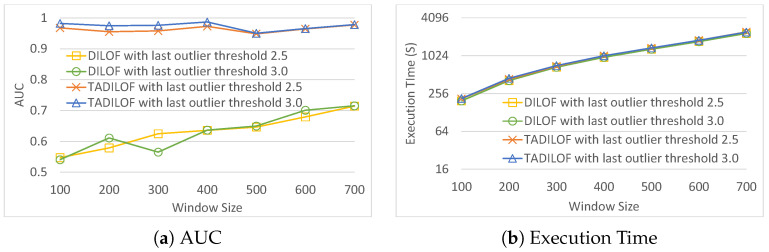
AUC and execution time on KDD 99 HTTP dataset using skipping scheme.

We set the threshold of last outlier to T={2.5,3.0} with the number of neighbors set at 8, and a window size of W={100,200,300,400,500,600,700}. The experimental results obtained using the HTTP KDD Cup 99 dataset are presented in Figure 9. Figure 9 illustrates that the modified skipping scheme achieved an AUC of more than 0.9 on the HTTP KDD Cup 99 dataset, regardless of the window size.

## 5. PM2.5 Sensors Case Study

In this section, we introduce the application of our proposed method that we used for monitoring air quality in Taiwan. There are several recent studies which have focused on air quality and PM2.5 forcasting [29,30,31,32], and anomaly detection in air quality [33].

In an effort to control air pollution in Taiwan, low-cost devices have been developed for monitoring air quality. These devices are referred to as LASSs. The Taiwanese government has initiated a project in cooperation with Edimax for the wide-scale deployment of LASS in elementary schools, high schools, and universities. The LASS used in this project are referred to as AirBox devices. Our objective in this study was to enable the real-time monitoring of all 2000 AirBox devices simultaneously.

We deployed a system in Taiwan for the detection of outliers in a large-scale dataset from PM2.5 sensors. This system provided 2000 data streams from 2000 sensors transmitting reading data at intervals of 5 min. The proposed method was used to detect outliers in each of the streams, with a focus on temporal outliers to compensate for inter-device variation in terms of quality and sensitivity. Following the identification of temporal outliers, we combined the positions of the devices with meteorological data to facilitate the detection of pollution events.

In addition, we used precision PM2.5 stations which are provided by the Environmental Protection Administration (EPA), Taiwan, to predict air quality. We integrated the data from precision PM2.5 sensors provided by EPA, Taiwan, because the quality of the data from precision PM2.5 sensors is better. However, there are only 77 PM2.5 stations in Taiwan and they provide an average PM2.5 value every hour. In this situation, we cannot find small pollution events. Therefore, we used low cost but large-scale PM2.5 devices for detecting pollution events. There are some advantages to using those PM2.5 sensors. The first benefit is that we can monitor air quality of Taiwan by a fine resolution on space because the number of active devices is more than 2000 regarding those that are deployed in Taiwan. The second benefit is that their sampling rate is 5 min. Therefore, we can also have a fine resolution on time domain to monitor air quality of Taiwan.

After getting fine resolution data based on both time and space, the challenge is how to use those data to detect pollution events. There are three challenges of using those data to detect pollution events. The first one is those devices are low cost and there is lack of maintenance. In general case, those kind of sensors need device correction every few month, so that the reading number is more accurate. The second challenge is there are numerous devices, and each device has a very high sampling rate which is every 5 min. We can see one of these devices as a data stream, and hence there are 2000 data streams. Therefore, we need to handle this large amount of data streams. Our proposed method has the capability to not only find outliers on different devices but also to deal with large number of data streams which have the high sampling rate. Next, we introduce how our method finds the pollution events in the following subsection.

### Monitoring a PM2.5 Pollution Event

In this section, we introduce how to use the proposed method to monitor PM2.5 pollution event. First, we define spatial neighbors of devices using average wind speed of Taiwan. According to Central Weather Bureau (CWB) of Taiwan, the average wind speed of Taiwan is 3.36 km/h. Therefore, we define neighbor distance to be 1.5 km, which means any two devices are neighbors if the distance between these two devices is less than 1.5 km.

Each device produces a data stream because every device samples the concentration of PM2.5 at interval of every five minutes and the number of data values is unbounded. We implemented our proposed method on each data stream. Thus, we can detect outliers on different devices separately. We call this type of outlier a temporal outlier because such outliers are compared with historical data points from the same device. If proposed method detects any temporal outlier on devices, we add the device to a set called outlier-event-pool and set an expire time as 30 min. In next 30 min, if we can find two neighbor devices in the outlier-event-pool for any device in the outlier-event-pool, we call this event a pollution event. Otherwise, it represents a spatial outlier of the device.

**Figure 10:**
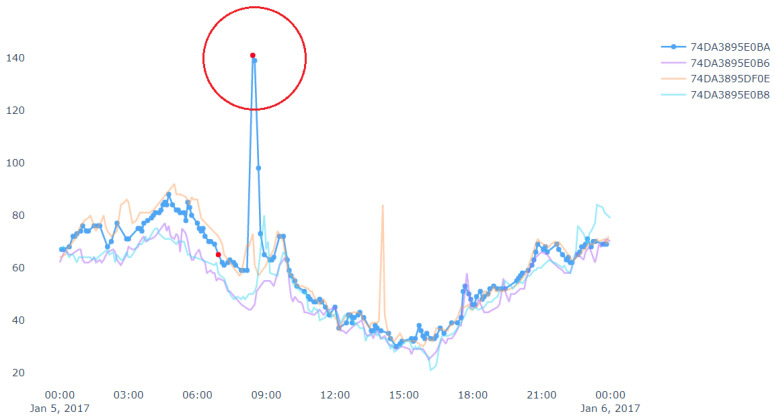
An example of a spatial (and temporal) outlier.

**Figure 11:**
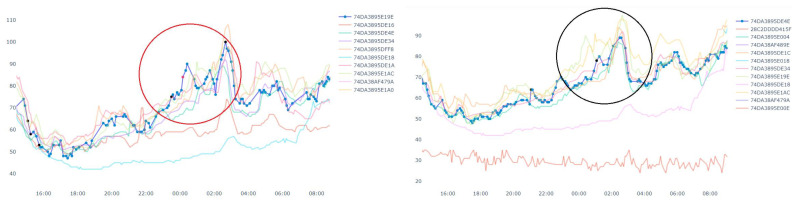
An example of a pollution event.

Figure 10 shows an example of spatial outlier. A spatial outlier means that there is only one device which has a sudden rise/fall in the measurement value and other nearby devices do not have any such change in the measurement. In Figure 10, the data stream in blue represents a target device which shows outlier data points marked in red. Outliers from other data streams are not shown, i.e., not marked in red in this figure. Similarly, Figure 11 shows an example of pollution event. At the left side of the figure, the measurement value from a device has a sudden rise. Then the neighbor devices in the right side of the figure also has a rise in the measurement value in next few minutes. Since this event may have been started by nearby device shown in the left side of the figure. Thus, we can get the potential pollution event region.

**Figure 12:**
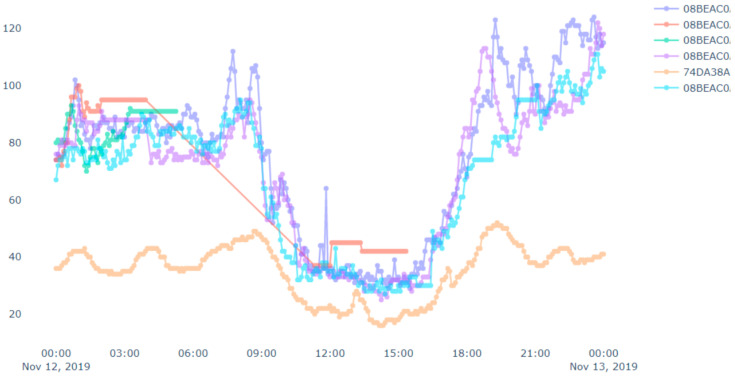
A case study of a fire event with PM2.5 sensors’ data.

Now, we discuss a use case related to a fire event, where we applied the proposed approach discussed above. In this case study, we targeted to track the pollution events where there is sudden increase in PM2.5 values. Our analysis targeted a fire event, which was reported at 17:51 2019/11/12 in Tainan city following reports of burning rubber. The Tainan EPB sent emergency notifications to Tainan citizens at 21:00. However, our system detected (and reports) the event at approximately 17:00. Figure 12 presents PM2.5 data for all devices in the vicinity of the fire throughout the day. We can see some flat lines in the readings. These are due to device malfunctions or reading errors (we have mentioned above about the issues related to the low-cost airbox devices). Similarly, we can see some bottom curves in Figure 11 and Figure 12. These are there because of the placements of the airbox devices. Some of the devices were placed indoors whereas other devices were placed outdoors. The indoor devices had a different environment (such as air conditioned room) than the outdoor devices, which affected the readings among different devices. Therefore, bottom curves are different from the others.

**Figure 13:**
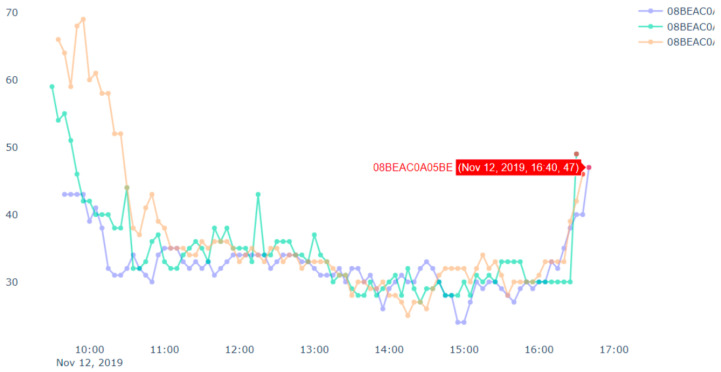
A case study on a fire event with PM2.5 sensors’ data—detected event.

Figure 13 shows the result that our implemented system detects the pollution event (fire event). In Figure 13, we can see that the proposed system sends the alert to subscribers at approximately 5 p.m.

## 6. Conclusions

This paper presents a novel algorithm to detect local outliers in data streams using LOF score. In addition, we used a time indicator with data points to resolve the issue of concept drift in data streams with the aim of improving accuracy in the detection of outliers. Moreover, we developed a novel method by which historical information is used to calculate approximate LOF values to improve accuracy with only a negligible increase in memory cost. The results of experiments illustrate that the proposed method, TADILOF, outperforms the state-of-the-art competitors in terms of AUC in most of the cases on various datasets. In addition, a practical application of the proposed scheme to PM2.5 sensor data clearly demonstrated its efficacy.

## Figures and Tables

**Table 1:** Datasets.

Dataset	# Data Points	# Dimensions	# Outlier Data Points	Need to Shuffle
Annthyroid	7200	6	534	false
Cardio	1831	21	176	true
HTTP (KDD Cup 99)	567,498	3	2211	false
Letter Recognition	1600	32	100	true
Mnist	7603	100	700	true
Musk	3062	166	97	true
Pendigits	6870	16	156	false
Satellite	6435	36	2036	false
SMTP (KDD Cup 99)	95,156	3	30	false
Vowels	1456	12	50	true

**Table 2:** Precision, recall, and F1 score on Annthyroid dataset.

Window Size	Precision			Recall			F1 Score		
	DILOF	TADILOF	MILOF	DILOF	TADILOF	MILOF	DILOF	TADILOF	MILOF
100	0.259074	0.224178	0.2289622	0.350187	0.383895	0.3506741	0.188322	0.198476	0.1945009
120	0.264844	0.222732	0.2331385	0.355993	0.392697	0.3582022	0.191396	0.200518	0.1975793
140	0.259869	0.213771	0.2369482	0.367790	0.404307	0.3630711	0.195014	0.201042	0.2018931
160	0.257486	0.218562	0.2381993	0.378652	0.415730	0.3679961	0.197863	0.206054	0.2023676
180	0.258819	0.217542	0.2464307	0.375094	0.418352	0.3726779	0.196032	0.207163	0.2043856
200	0.264608	0.218433	0.2433799	0.380899	0.426779	0.3750937	0.199770	0.210723	0.2032031

**Table 3:** Precision, recall, and F1 score on Cardio dataset.

Window Size	Precision			Recall			F1 Score		
	DILOF	TADILOF	MILOF	DILOF	TADILOF	MILOF	DILOF	TADILOF	MILOF
100	0.3467338	0.3693657	0.3151908	0.3914205	0.4547727	0.3050568	0.2156009	0.2700938	0.1910918
120	0.3381342	0.3508179	0.3284806	0.3751136	0.4397159	0.3127273	0.2011454	0.2549688	0.1956810
140	0.3218308	0.3431242	0.3028065	0.3655682	0.4323864	0.2982955	0.1903846	0.2475356	0.1816338
160	0.3151459	0.3354626	0.3063062	0.3569886	0.4157387	0.3037500	0.1832554	0.2353132	0.1818394
180	0.3209461	0.3262367	0.3021858	0.3512500	0.4147726	0.3022158	0.1781800	0.2318135	0.1784863
200	0.3120879	0.3206106	0.2919695	0.3422159	0.4043182	0.2994319	0.1706907	0.2229609	0.1725868

**Table 4:** Precision, recall, and F1 score on Letter Recognition dataset.

Window Size	Precision			Recall			F1 Score		
	DILOF	TADILOF	MILOF	DILOF	TADILOF	MILOF	DILOF	TADILOF	MILOF
100	0.12697568	0.11241224	0.2220374	0.2059	0.2308	0.2593	0.06782202	0.08080138	0.1311881
120	0.14821722	0.16318930	0.2436584	0.2139	0.2443	0.2616	0.07340590	0.09222663	0.1351141
140	0.15472405	0.15568830	0.2298457	0.2193	0.2517	0.2618	0.07528581	0.09592009	0.1339924
160	0.17106840	0.17378370	0.2574958	0.2271	0.2625	0.2663	0.08395074	0.10338267	0.1392814
180	0.19773730	0.20144530	0.2839139	0.2335	0.2706	0.2718	0.08891001	0.11030273	0.1452346
200	0.20078190	0.19852930	0.2843155	0.2375	0.2732	0.2696	0.09236163	0.11257103	0.1431587

**Table 5:** Precision, recall, and F1 score on Mnist dataset.

Window Size	Precision			Recall			F1 Score		
	DILOF	TADILOF	MILOF	DILOF	TADILOF	MILOF	DILOF	TADILOF	MILOF
100	0.221380000	0.191549667	0.240385667	0.209047667	0.243714000	0.241381000	0.074075133	0.107531667	0.135068000
120	0.200322333	0.272608333	0.254099000	0.212190333	0.248285667	0.242285667	0.076509233	0.112503333	0.138804667
140	0.198687667	0.260456000	0.292730000	0.216428333	0.257047667	0.249047667	0.080405100	0.121062667	0.140650667
160	0.228525333	0.293933667	0.301560333	0.217190333	0.262143000	0.252381000	0.080920167	0.125967000	0.144668000
180	0.177270000	0.281288667	0.307995000	0.219428667	0.265905000	0.257190333	0.083189067	0.127457667	0.145774667
200	0.186638333	0.297026333	0.298204333	0.221857000	0.270571333	0.257428333	0.084071200	0.133201333	0.147606667

**Table 6:** Precision, recall, and F1 score on Musk dataset.

Window Size	Precision			Recall			F1 Score		
	DILOF	TADILOF	MILOF	DILOF	TADILOF	MILOF	DILOF	TADILOF	MILOF
100	0.4421690	0.4141334	0.4397151	0.3313403	0.5407216	0.2925772	0.2013681	0.3326063	0.1927423
120	0.4083079	0.4027774	0.4104153	0.2854639	0.4829896	0.2662887	0.1614765	0.2968274	0.1652040
140	0.3923583	0.3881677	0.4092076	0.2637113	0.4541238	0.2397939	0.1438728	0.2802120	0.1452295
160	0.3857042	0.3906538	0.3659905	0.2461858	0.4086599	0.2360825	0.1276172	0.2505055	0.1363073
180	0.4097445	0.3819757	0.3576605	0.2322681	0.3759795	0.2064948	0.1189272	0.2324761	0.1081695
200	0.3928690	0.3710396	0.3543731	0.2198969	0.3363918	0.1968042	0.1107008	0.2034848	0.1058940

**Table 7:** Precision, recall, and F1 score on Pendigits dataset.

Window Size	Precision			Recall			F1 Score		
	DILOF	TADILOF	MILOF	DILOF	TADILOF	MILOF	DILOF	TADILOF	MILOF
100	0.0540172	0.0955094	0.10309758	0.312179	0.445513	0.3918589	0.0699342	0.103071	0.11157027
120	0.0517353	0.1142970	0.08978204	0.322436	0.483333	0.3849999	0.0718471	0.111540	0.10553305
140	0.0582843	0.0868875	0.08765809	0.331410	0.481410	0.3944872	0.0731726	0.108052	0.10599671
160	0.0553464	0.0676196	0.07356239	0.330128	0.485897	0.3857051	0.0716655	0.104400	0.09873375
180	0.0429529	0.0734877	0.07456543	0.314103	0.478205	0.3844233	0.0598091	0.105238	0.09594913
200	0.0500194	0.0743026	0.08276458	0.328205	0.484615	0.3871795	0.0667915	0.102561	0.09896692

**Table 8:** Precision, recall, and F1 score on Satellite dataset.

Window Size	Precision			Recall			F1 Score		
	DILOF	TADILOF	MILOF	DILOF	TADILOF	MILOF	DILOF	TADILOF	MILOF
100	0.486230	0.488270	0.4720359	0.256925	0.333792	0.2466356	0.229341	0.303750	0.2279936
120	0.498198	0.496403	0.4636664	0.257122	0.341945	0.2488359	0.228337	0.307481	0.2281764
140	0.481029	0.494004	0.4694753	0.260806	0.332760	0.2601866	0.230814	0.295530	0.2381250
160	0.498065	0.492069	0.4886004	0.266994	0.325688	0.2682712	0.233976	0.286045	0.2438489
180	0.498793	0.507865	0.4879055	0.278340	0.339096	0.2791945	0.242351	0.298429	0.2519019
200	0.491820	0.505140	0.4673425	0.289293	0.341454	0.2788359	0.252402	0.297941	0.2501888

**Table 9:** Precision, recall, and F1 score on SMTP dataset.

Window Size	Precision			Recall			F1 Score		
	DILOF	TADILOF	MILOF	DILOF	TADILOF	MILOF	DILOF	TADILOF	MILOF
100	0.00265890	0.00164838	0.002386766	0.7633	0.7400	0.5029	0.00525291	0.00327270	0.004681796
200	0.00256851	0.00247500	0.002520710	0.7733	0.7900	0.5147	0.00507621	0.00491061	0.004933168
300	0.00332737	0.00344192	0.002814311	0.7933	0.8133	0.6179	0.00655182	0.00679199	0.005521916
400	0.00265894	0.00238982	0.002669190	0.7867	0.9133	0.6603	0.00525692	0.00475252	0.005260103
500	0.00191050	0.00313257	0.002218161	0.7467	0.9467	0.6417	0.00379399	0.00620751	0.004383276
600	0.00203202	0.00191777	0.001829777	0.7700	0.9767	0.5839	0.00403406	0.00382331	0.003621248
700	0.00168554	0.00185686	0.001824417	0.6767	0.9067	0.5933	0.00334839	0.00369957	0.003614118

**Table 10:** Precision, recall, and F1 score on Vowels dataset.

Window Size	Precision			Recall			F1 Score		
	DILOF	TADILOF	MILOF	DILOF	TADILOF	MILOF	DILOF	TADILOF	MILOF
100	0.14336408	0.1570996	0.1922093	0.3256	0.3898	0.4302	0.1121199	0.130352	0.179416
120	0.16889650	0.1551854	0.1959132	0.3476	0.4350	0.4202	0.1239128	0.148712	0.171690
140	0.16830130	0.1644227	0.2006647	0.3660	0.4604	0.4350	0.1329999	0.158371	0.175122
160	0.17210987	0.1958837	0.2394359	0.3756	0.4758	0.4384	0.1360716	0.166075	0.179563
180	0.16043631	0.1741156	0.2275521	0.3862	0.5022	0.4494	0.1367308	0.174471	0.182075
200	0.16436960	0.1809000	0.2074316	0.3914	0.5000	0.4348	0.1390244	0.173421	0.173099
